# The Quack Question as Handled by the City Council of Mansfield, O.

**Published:** 1893-04

**Authors:** 


### The Quack Question as Handled by the City Council of Mansfield, O.

At the meeting of the Council of the city of Mansfield, O., on November 29, 1892, an ordinance was passed by a two-thirds majority, which prevents any quacks or itinerant venders of medicine, "tooth-pullers" or other impostors practicing their nefarious schemes in that city without first getting a permit from the Health Officer, who, by the ordinance, is required to be a regular physician. The ordinance also requires these quacks to display a diploma from some respectable college before the Health Officer can give them the necessary certificate entitling them to a license at all. On the presentation of said certificate to the Mayor they can receive a license for which they must pay not less than twenty-five dollars, nor more than fifty dollars a day, and are also subject to a fine of not less than twenty-five nor more than fifty dollars, for each and every offense, for the violation of this ordinance.

The law goes into effect immediately after its publication, and covers physicians, mid-wives, pharmacists and dentists. If every City Council throughout the State of Ohio would follow the example set by the Council of Mansfield, they would take a grand step in the direction of getting rid of quacks and impostors which infest all our large cities. This plan has been tried in Kentucky, and so far has proved to be of great advantage in getting rid of these leeches, and should be followed by all the states that have no special laws or that cannot get special legislation to remedy this great evil.

